# The physiological characteristics of inward rectifying potassium channel Kir4.2 and its research progress in human diseases

**DOI:** 10.3389/fcell.2025.1519080

**Published:** 2025-04-24

**Authors:** Hongling Zhang, Zhongyuan Bai, Yanfeng Xi

**Affiliations:** ^1^ Pathology Department, The Cancer Hospital Affiliated to Shanxi Medical University, Taiyuan, China; ^2^ Colorectal Surgery, The First Clinical Medical College of Shanxi Medical University, Taiyuan, China; ^3^ Pathology Department, Shanxi Cancer Hospital, Taiyuan, China

**Keywords:** KCNJ15, inward rectifying potassium channel, diseasa, physiological characteristic, cancer

## Abstract

Kir4.2 is a member of the inward rectifying potassium channel family, encoded by the KCNJ15 gene. The Kir4.2 protein is expressed in various organs including the kidneys, liver, pancreas, bladder, stomach, and lungs. Kir4.2 not only forms functional homomeric channels, but also heteromeric channels with Kir5.1. An increasing number of studies indicate that the function of the Kir4.2 channel should not be underestimated. Kir4.2 participates in cell electrotaxis chemotaxis by sensing extracellular electric fields and functions as a K + sensor in the proximal tubules of the kidney, playing a crucial role in maintaining acid-base and potassium balance. This article provides a comprehensive review of the main physiological characteristics of the Kir4.2 channel, the various pathological processes it is involved in, and the human diseases resulting from Kir4.2 dysfunction.

## 1 Introduction

The ion channels of biological membranes mediate the passive transport of various inorganic ions across the membrane, and the permeability of biological membranes to ions is closely related to various life processes. Ionic channels are composed of special proteins produced by cells, which aggregate and embed on the cell membrane, and regulate the entry and exit of corresponding substances through the opening and closing of channels. According to the activation mechanism, ion channels can be divided into: 1) voltage-gated channels, whose opening is controlled by membrane potential, such as Na^+^, Ca^2+^, Cl^−^, and some K^+^ channels; 2) Chemical gated channels, which are activated by the interaction between chemicals and membrane receptors, such as Ach receptor channels, amino acid receptor channels, Ca^2+^ activated K^+^channels, etc.,; 3) Mechanical gated channels, which are activated and deactivated by local mechanical stimuli on the membrane, such as sensory nerve endings, auditory hair cells, endothelial cells on blood vessel walls, and skeletal muscle cells. K^+^ channels are a type of protein complex that exists on biological membranes and has a certain selective permeability to K^+^. By controlling the dynamic balance of K^+^ inside and outside the cell, they regulate the cell membrane potential and participate in a series of physiological or pathological processes. According to channel characteristics, K^+^ channels can be divided into: 1) voltage-gated K^+^ channels; 2) inward rectifying K^+^ channel; 3) two-pore-domain K^+^ channel; 4) Ca^2+^-activated K^+^ channels; And 5) delay K^+^ channel.

**FIGURE 1:**
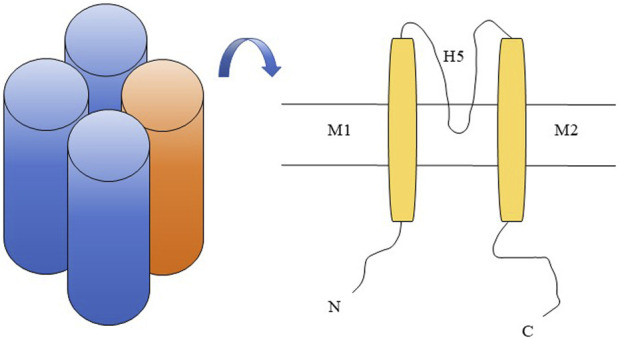
Kir4.2 channel schematic diagram. Kir4.2 is composed of four subunits (left in the figure), with each subunit comprising a highly conserved pore (P) region that harbors a conserved H5 segment, as well as two transmembrane domains, M1 and M2, situated on either side of the pore region (right in the figure).

The first inward rectifying potassium (Kir) channel gene was reported by Kubo et al. (1993). The characteristic of the Kir channel is that the conductivity increases during membrane potential hyperpolarization and decreases during depolarization. And this phenomenon is due to the fact that Mg^2+^ and other high valence ions inside the cell move towards the inner opening of the channel and block it when the membrane potential is depolarized, making it easier for K^+^ to flow inward through the Kir channel than out. This characteristic makes it play an important role in maintaining cell resting membrane potential, regulating cell excitability, and maintaining potassium homeostasis (Jan and Jan, 1997; Doupnik et al., 1995; Hibino et al., 2010). The general structure of a Kir channel consists of four subunits, each consisting of a highly conserved pore (P) region and two transmembrane domains (M1 and M2) located on either side (Figure 1). The P region contains a conserved H5 fragment, while the H5 and M2 segments bind to the carboxyl end hydrophilic domain, which is crucial for potassium permeation (Abraham et al., 1999). The currently known Kir family mainly consists of seven subfamilies (Kir1. x-Kir7. x), which can be divided into four groups based on their functions: 1) classical Kir channel: Kir2. x; 2) G-protein coupled Kir channel: Kir3. x; 3) ATP sensitive Kir channel: Kir6. x; 4) Kir channels affected by intracellular pH: Kir1. x, Kir4. x, Kir5. x, and Kir7. x (Hibino et al., 2010).

**FIGURE 2:**
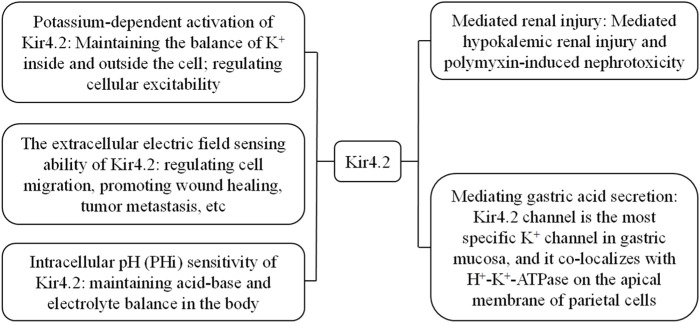
The main physiological characteristics and related pathological processes of Kir4.2.

Gosset et al. (1997) first revealed the existence of the gene KCNJ15 encoding the Kir4.2 (also known as IRKK and Kir1.3) channel in the Down syndrome chromosome one region (DCR1) on chromosome 21. Previous studies have demonstrated that Kir4.2 protein is expressed and performs corresponding functions in human kidneys, lungs, and pancreas (Shuck et al., 1997). Kir4.2 plays an important role in human physiological processes (Figure 2), such as the interaction between Kir4.2 channels and polyamines inducing extracellular electric fields to induce directional migration of cells (Nakajima and Zhao, 2016), and Kir4.2 is necessary for histamine stimulated gastric acid secretion (He et al., 2011). With further researchs, it has been found that Kir4.2 can mediate low potassium induced kidney injury and polymyxin induced nephrotoxicity (Terker et al., 2022; Lu et al., 2022). In addition, KCNJ15 gene mutation is related to type 2 diabetes, Alzheimer’s disease, epilepsy and other diseases (Okamoto et al., 2010; Zhou et al., 2018; Wang et al., 2022). In cancer research, KCNJ15 gene participates in the cancer process as a differential gene in a variety of cancers, such as kidney cancer, esophageal squamous cell carcinoma, breast cancer and glioma (Liu et al., 2019; Nakamura et al., 2020; Qiao et al., 2023; Veeravalli et al., 2012). What is even more impressive is that Kir4.2 protein is expressed in retinal pigment epithelial cells and plays a role in maintaining the survival and proliferation ability of retinal pigment epithelial cells (Beer et al., 2022).

With the exploration of the role of Kir4.2 channel in human diseases, we are more certain that it still has many important functions worth further exploration. In summary, this article will review the research progress of Kir4.2 channel from three aspects: the main physiological characteristics of Kir4.2 channel, the pathological processes it participates in, and its connection with human diseases. The aim is to provide new ideas for further exploring the biological functions of Kir4.2 channel and its potential value in human diseases.

## 2 Main physiological characteristics of Kir4.2

### 2.1 Potassium dependent activation of Kir4.2

As is well known, Kir channels are involved in regulating cellular excitability and K^+^ transport processes. In the Kir subfamily, the activation of Kir1.1 (Dahlmann et al., 2004; Doi et al., 1996), Kir4.1 (Edvinsson et al., 2011a), and Kir4.2 (Pearson et al., 1999) depends on the extracellular K^+^ concentration ( $K_{0}^{+}$ ). $K_{0}^{+}$ participates in the regulation of Kir4.2 channels by affecting the number of cell surface channels or altering the properties of channels present on the plasma membrane surface (Hoshi et al., 1991). Edvinsson et al. (2011b) found that Kir4.2 is sensitive to changes in $K_{0}^{+}$ , and this characteristic is completely eliminated when Kir4.2 is co expressed with Kir5.1. Unlike Kir1.1, the introduction of mutation (K66M) significantly reduced the pHi sensitivity of Kir4.2 channel, while the sensitivity to $K_{0}^{+}$ remained unchanged, indicating that there is no coupling between pHi sensitivity and $K_{0}^{+}$ sensitivity of Kir4.2. That is to say, the sensitivity of Kir4.2 to $K_{0}^{+}$ is independent of pHi. Based on the kinetic model, Kir4.2 exists in at least three states on the plasma membrane: the inactive state, the intermediate unstable state, and the active state. The intermediate unstable state is also referred to as the $K_{0}^{+}$ -sensitive state. The transition of Kir4.2 channels from the inactive state to the active state initially occurs at a relatively slow rate and happens independently of $K_{0}^{+}$ in a random manner. Once entering the intermediate unstable state, $K_{0}^{+}$ interacts with the channel, facilitating its rapid transition to the active state (Edvinsson et al., 2011b). Given the localization of Kir4.2 in the kidney, this potassium-dependent activation suggests that Kir4.2 can act as a potassium sensor to maintain K^+^ balance.

### 2.2 The extracellular electric field induction function of Kir4.2

The directed migration of cells is of great significance in various physiological and pathological processes such as embryonic development, angiogenesis, wound repair, inflammatory response, and tumor metastasis (Cortese et al., 2014; Espina et al., 2022). The directional migration of cells guided by extracellular electric fields is called electrotaxis. Currently, there are multiple types of cells have directional migration by induction of extracellular electric field, such as corneal epithelial cells, keratinocytes, endothelial cells, lymphocytes, stem cells, and some tumor cells (McCaig et al., 2005). For example, during wound healing, trans-epithelial potentials (TEPs) are disrupted, with the wound edge becoming an anode and the wound center becoming a cathode. The resulting endogenous electric field guides the migration of epidermal cells to promote wound healing (Liu et al., 2024; Zhao, 2009; Yang et al., 2022). Extracellular electric fields not only regulate cell migration, but also play an essential role in cell proliferation, localization, and polarization processes (Cortese et al., 2014; Zhao et al., 1996; Clancy et al., 2021). Ion channels play a crucial role in the generation and induction of biological currents. Studies have shown that knocking down KCNJ15 significantly reduces the electrotaxis of cells in the extracellular electric field, and the migration speed is the same as that of non target siRNA control cells or cells without an extracellular electric field, indicating that knocking down KCNJ15 has a specific effect on the orientation induction of cells in the extracellular electric field (Nakajima et al., 2015).

Polyamines are a class of organic compounds containing two or more amino groups. The primary raw materials for their synthesis are ornithine and arginine. The most prevalent polyamines with significant physiological functions are putrescine (PUT), spermidine (SPD), and spermine (SPM). They play a crucial role in regulating nucleic acid and protein structures, protein synthesis, interactions between proteins and nucleic acids, oxidative balance, and cell proliferation (Zahedi et al., 2022; Weiger and Hermann, 2014). The inward rectification characteristics of Kir channels are mediated by intracellular polyamines. Positively charged intracellular polyamines bind to negatively charged amino acid residues located in the pore region of Kir channels, preventing the outward flow of K^+^ (Hibino et al., 2010; Ficker et al., 1994). Polyamine depletion alters the inward rectification characteristics of Kir channels, causing K^+^ to flow reversely outward (Shyng et al., 1996). Using the polyamine analog N1, N11-diethylnorspermine (DENSPM) to deplete intracellular polyamines, DENSPM treatment completely eliminated electrotaxis, and cells exhibited random migration. Culturing cells with PUT (an important precursor for SPM/SPD synthesis) significantly increased intracellular polyamine concentration, enhancing cell electrotaxis. However, knocking out KCNJ15 completely eliminated the PUT-induced enhancement of electrotaxis (Nakajima et al., 2015), suggesting that Kir4.2 senses extracellular electric fields through interaction with polyamines. By further constructing a polyamine-binding deficient KCNJ15 mutant, it was observed that this mutant significantly reduced the cell orientation in an extracellular electric field, yet it did not affect cell motility (Nakajima and Zhao, 2016; Nakajima et al., 2015). This further demonstrates that the interaction between Kir4.2 protein and intracellular polyamines is essential for cell sensing of extracellular electric fields. In conclusion, Kir4.2 achieves directional sensing of extracellular electric fields through interaction with polyamines. This mechanism may facilitate the exploration of more potential values of Kir4.2 channels in human disease research.

### 2.3 Intracellular pH (pHi) sensitivity of Kir4.2

In 1997, Shuck et al. (1997) screened and identified two Kir-related cDNAs, Kir1.3 and Kir1.2, from a human kidney cDNA library. The amino acid sequences of the two share 62% homology, but compared to Kir1.2, Kir1.3 does not express a functional channel in *Xenopus laevis* oocytes. Conversely, Pearson et al. (1999) demonstrated that Kir4.2 exhibits strong inward rectification characteristics in *Xenopus laevis* oocytes, and intracellular acidification can reversibly reduce the current of Kir4.2 channels. Research has shown that Kir4.2 has significantly higher inherent pHi sensitivity than Kir4.1, which is due to its presence of a C-terminal pHi-sensitive mechanism (Pearson et al., 2006; Pessia et al., 2001).

Kir5.1, encoded by KCNJ16, is expressed in many organs and tissues (Liu et al., 2000). Previous studies have shown that Kir5.1 does not form a functional channel, but can form functional heteromeric channels with Kir4.2 and Kir4.1 channels (Kir4.2/Kir5.1 and Kir4.1/Kir5.1). The presence of the pHi-sensitive mechanism at the C-terminus of Kir4.2 does not significantly increase the sensitivity of Kir4.2/Kir5.1 to pHi (Pearson et al., 1999; Pessia et al., 2001; Tanemoto et al., 2000; Xu et al., 2000; Yang et al., 2000). By expressing Kir4.2 and Kir4.2/Kir5.1 fusion proteins in HEK293 cells, it was found that Kir5.1 can sensitize Kir4.2 to intracellular Mg^2+^, polyamines, and intracellular PIP2 levels (Lam et al., 2006). The pHi sensitivity of the Kir4.2 channel and its localization in renal tubular epithelial cells suggest that it may be involved in the regulation of acid-base and electrolyte balance in the kidney.

## 3 Pathological processes associated with Kir4.2

### 3.1 The role of Kir4.2 in the process of kidney injury

Kir4.2 mediates low potassium-induced renal injury. The proximal tubule (PT) is the primary site for ammonia production, gluconeogenesis, and reabsorption of primary urine in the kidney, and it is also the main target of renal injury. In mouse kidneys, Kir4.2 and Kir5.1 are located on the basolateral membrane in the form of heterotetramers (Schlingmann et al., 2021), and hyperchloremia acidosis, reduced threshold for bicarbonate reabsorption, and decreased urinary NH4^+^ can be observed in KCNJ15^−/−^ mice (Bignon et al., 2020). Studies have shown that low potassium diet or low blood potassium caused by increased aldosterone can induce specific renal injury, which is dependent on the proximal tubule Kir4.2 channel (Terker et al., 2022). It is well known that renal ammonia metabolism is crucial for maintaining acid-base balance, and the proximal tubule is the main site for ammonia production (Weiner and Verlander, 2013). Under low potassium conditions, the Kir4.2 channel mediates the efflux of potassium from the basolateral side of the proximal tubule, causing intracellular acidosis, further activating the phosphate-dependent glutaminase-catalyzed ammonia production pathway, and ultimately leading to renal injury (Terker et al., 2022). Furthermore, under low potassium conditions, the Kir4.2 channel promotes the activation of the mTOR/AKT signaling pathway in proximal tubular cells, thereby regulating the kidney’s response to low potassium signals and maintaining K^+^ balance (Zhang Y. et al., 2024). Therefore, the Kir4.2 channel in the kidney serves as a potassium sensor in the proximal tubules to maintain K^+^ balance and mediates low potassium-induced renal injury, suggesting that Kir4.2 holds potential value in the clinical treatment of low potassium-induced renal injury.

Kir4.2 mediates polymyxin-induced nephrotoxicity. Polymyxins, a group of polypeptide antibiotics produced by polymyxa bacteria, exhibit inhibitory effects on most Gram-negative bacteria, and polymyxin-induced nephrotoxicity is a significant factor leading to poor treatment outcomes. Previous studies have shown that polymyxins accumulate significantly in renal tubular epithelial cells (Azad et al., 2015b), subsequently inducing nephrotoxicity through pathways such as apoptosis, mitochondrial damage, endoplasmic reticulum stress, oxidative stress, and inhibition of the cell cycle (Azad et al., 2019; Azad et al., 2015a). Additionally, polymyxins bind to Kir4.2, disrupting K^+^ homeostasis and inducing cell membrane depolarization by increasing the open state of Kir4.2 channels, ultimately leading to nephrotoxicity. Knocking out KCNJ15 or KCNJ16 in human renal tubular HK-2 cells individually can attenuate polymyxin-induced membrane depolarization, reduce polymyxin accumulation in cells, and significantly enhance resistance to polymyxin-induced toxicity (Lu et al., 2022). Thus, it is evident that polymyxins induce nephrotoxicity by directly binding to the Kir4.2/Kir5.1 heterotetramer. Therefore, in the clinical application of polymyxins, Kir4.2 and Kir5.1 inhibitors may serve as a strategy to reduce nephrotoxicity.

### 3.2 Kir4.2 mediates histamine-stimulated gastric acid secretion

The secretion of gastric acid by parietal cells is mediated by proton pumps (H^+^-K^+^-ATPase) and H^+^-HCO_3_^−^ exchangers. Under the stimulation of histamine and other factors, the proton pump consumes ATP to decompose water molecules and transports H^+^ into the lumen at a ratio of 1:1 in exchange for K^+^. The continuous supply of K^+^ in the lumen relies on K^+^ channels. Meanwhile, a large amount of residual OH^−^ in the cell, under the action of carbonic anhydrase, converts to HCO3^-^ together with CO_2_. Subsequently, under the action of H^+^-HCO_3_^-^ exchangers, it flows into the capillaries on the gastric wall side of the parietal cells, absorbs Cl^−^ from the capillaries into the cell, and discharges it into the gastric lumen through Cl^−^ channels. Finally, it combines with hydrogen ions discharged into the gastric lumen to form hydrochloric acid (Engevik et al., 2020). Obviously, the secretion of gastric acid requires a continuous supply of K^+^. Previous studies have shown that KCNQ1 (Song et al., 2009; Nguyen et al., 2013; Lambrecht et al., 2005), KCNJ1 (Vucic et al., 2015), KCNJ2 (Malinowska et al., 2004), KCNJ10 (Song et al., 2011), and KCNJ15 (He et al., 2011; Ficker et al., 1994) are all related to gastric acid secretion.

The results of the qRT-PCR experiment showed that the Kir4.2 channel is the most specific K^+^ channel in gastric mucosa. Western blot analysis further confirmed the abundant presence of Kir4.2 protein in gastric mucosa. Immunofluorescence staining indicated that Kir4.2 protein is expressed in both gastric parietal cells and chief cells. In resting parietal cells, Kir4.2 is mainly located in the cytoplasm. In resting parietal cells, Kir4.2 is primarily located in the cytoplasm. However, upon histamine stimulation, Kir4.2 undergoes translocation and co-localizes with H^+^-K^+^-ATPase on the apical membrane of parietal cells (He et al., 2011). This observation is further corroborated by live-cell imaging systems. After infection with adenovirus carrying the KCNJ15 shRNA plasmid, the protein level of Kir4.2 decreases in primary rabbit gastric parietal cells, and these cells exhibit no response to histamine-induced acid secretion (Yuan et al., 2015). These findings suggest that the absence of KCNJ15 can lead to gastric acid secretion dysfunction.

## 4 Research progress of Kir4.2 in human diseases

### 4.1 Kir4.2 and type 2 diabetes

Type 2 diabetes (T2DM) is recognized as one of the major health issues in developed countries, and it is also becoming increasingly prevalent in developing countries. Numerous studies have revealed significant differences in the average BMI of T2DM patients among different populations (Zimmet et al., 2001; Davis et al., 2001; Sone et al., 2002). KCNJ15 is relatively highly expressed in the pancreas, especially more prominently in the Langerhans islets, and there is a significant correlation between the KCNJ15-related single nucleotide polymorphism (SNP) rs3746876-T and T2DM (Okamoto et al., 2010; Fukuda et al., 2013). Okamoto et al. (2010) found that the KCNJ15-related SNP rs3746876 was significantly associated with lean T2DM patients (BMI <24 kg/m^2^) in Asia, and the level of KCNJ15 mRNA in peripheral blood of patients with rs3746876-T was higher than that of patients with rs3746876-C. *In vitro* functional analysis showed that under high glucose concentration (25 mmol/L) conditions, overexpression of KCNJ15 reduced insulin secretion, but no significant changes were observed under normal blood glucose conditions. Interestingly, Fukuda et al. (2013) conducted a replication study on the correlation between the KCNJ15-related SNP rs3746876 and T2DM, and found that rs3746876-T was significantly associated with T2DM, but the direction of effect was opposite to that of previous studies, and this correlation was only significant in obese T2DM patients (BMI >24 kg/m^2^). Subsequently, Okamoto et al. (2012) further investigated the effect of KCNJ15 on insulin secretion, and found that the level of KCNJ15 mRNA in Langerhans islets of T2DM patients was significantly higher than that of non-diabetic controls, and high glucose concentration (25 mmol/L) could induce the expression of KCNJ15, while knockdown of KCNJ15 under the same conditions could increase insulin secretion *in vitro* and *in vivo*.

The Ca^2+^-sensing receptor (CaSR), a member of the G protein-coupled receptor (GPCR) superfamily, is closely related to systemic Ca^2+^ homeostasis. CaSR is expressed in neurons, oligodendrocytes, breast ductal epithelial cells, fibroblasts, and Langerhans islet cells, and it plays a role in insulin secretion in pancreatic islet β-cells (Rasschaert and Malaisse, 1999; Malaisse et al., 1999). Studies have shown that activation of CaSR triggers a significant but transient insulin secretion response in human pancreatic islet cells and insulin-secreting cells MIN6, and can enhance glucose-induced insulin secretion. Furthermore, the activation of CaSR is associated with the activation of p42/44 mitogen-activated protein kinase (MAPK) (Gray et al., 2006). In mouse kidney tissues, CaSR selectively interacts with Kir4.1 and Kir4.2, leading to channel inactivation and subsequently inhibiting electrolyte transport in nephrons (Huang et al., 2007). This interaction suggests that KCNJ15 may be involved in insulin regulation through its interaction with CaSR. Further research has confirmed that CaSR and KCNJ15 are co-expressed in rat insulinoma (INS1) cells. Gene double knockdown results indicate that inactivation of CaSR reduces insulin secretion. Moreover, in the absence of CaSR, inactivation of KCNJ15 does not increase insulin secretion, suggesting that CaSR is a necessary condition for KCNJ15 to affect insulin secretion (Okamoto et al., 2012). Additionally, based on our understanding of the function of the Kir family, KCNJ15 may negatively regulate insulin secretion by maintaining the resting membrane potential of pancreatic β-cells and inhibiting depolarization. In summary, KCNJ15 is a risk gene associated with T2DM. The emergence of contradictory conclusions may be due to differences inliving environments, racial, and/or regional factors, indicating that more research is needed to clarify the contribution of KCNJ15 to T2DM susceptibility.

### 4.2 Kir4.2 and neurological diseases

Alzheimer’s disease (AD) is a complex neurodegenerative disorder. With the aging of the population, its prevalence is rapidly increasing, making it one of the leading causes of death among the elderly (Scheltens et al., 2021). The pathogenesis of AD is complex, with genetics playing a pivotal role. A whole-genome sequencing study revealed common genetic risk factors for AD, including APOE, GCH1, and KCNJ15. Genotype-phenotype analysis revealed that the variation at the KCNJ15-related SNP rs928771 locus affects the age of onset of Alzheimer’s Disease (AD), with a small number of allele carriers experiencing earlier onset (Zhou et al., 2018). Through research on the relationship between the levels of immune-related plasma biomarkers and the genotype of rs928771, it was found that AD subjects exhibited a genotype-dependent reduction in various immune-related plasma biomarkers. This suggests that the KCNJ15 variant may affect the progression of AD by regulating the immune system (Zhou et al., 2018).

Epilepsy is one of the most common neurological diseases, caused by abnormal neuronal discharge in the brain, and characterized by its recurrence and complexity (Shorvon, 1990). K^+^ channels are involved in regulating neuronal excitability and play a crucial role in the membrane repolarization process of neurons. Therefore, K^+^ channels may play a role in the occurrence of epilepsy (Nikitin and Vinogradova, 2021). Mutations in the Kir family are relatively rare in epilepsy patients. Kir4.1 is mainly expressed in astrocytes, and its functional defects are associated with EAST (epilepsy, ataxia, sensorineural hearing loss, and renal tubular disorders) syndrome (Scholl et al., 2009). Mice lacking the GIRK2 gene (encoding Kir3.2 channel) exhibit spontaneous seizures and are more prone to pharmacological seizures induced by γ-aminobutyric acid antagonists (Signorini et al., 1997). A comprehensive bioinformatics analysis revealed that KCNJ15 is significantly downregulated in the brain tissue of the medial temporal lobe in patients with drug-resistant epilepsy. Protein-protein interaction (PPI) analysis indicated that the protein encoded by KCNJ15 directly interacts with the two epilepsy drug targets encoded by GABBR1 and GABBR2, further supporting the role of KCNJ15 in epilepsy. Expression quantitative trait locus (eQTL) analysis showed that the epilepsy-related SNP rs2833098 may become a risk marker for epilepsy by regulating the expression level of KCNJ15 in human temporal lobe brain tissue (Wang et al., 2022). It is evident that the Kir family is involved in the occurrence of epilepsy. As a potential biological target for epilepsy, KCNJ15 requires further experimental verification and more evaluations in different populations.

### 4.3 Kir4.2 and cancer

Cancer poses a significant public health issue globally, with its incidence and mortality rates steadily rising, thus emerging as the primary cause of human mortality (Siegel et al., 2022). At present, the main treatment methods of cancer include surgery, chemotherapy, radiotherapy and biological therapy. Given that ion channels are extensively involved in various cellular physiological and pathological processes, it is unavoidable for genes encoding these channels to be present during the oncogene transformation process (Prevarskaya et al., 2018). K^+^ channels are extensively distributed across various human tissues, playing a role in cell adhesion and migration, apoptosis and proliferation, cell cycle regulation, cell volume control, angiogenesis, and other processes pertinent to tumor biology. Consequently, the expression and dysfunction of K^+^ channels are closely linked to tumor progression (Pardo and Stühmer, 2014; Li et al., 2022). Evidently, K^+^ channels hold promise as markers for tumor diagnosis and prognosis, potentially offering novel insights for targeted tumor therapy.

The Kir family represents a class of non-voltage-gated K^+^ channels, playing a crucial role in maintaining cell resting membrane potential, regulating cell excitability, and modulating cell volume. KCNJ15 exhibits differential expression across various cancers. Currently, research on KCNJ15 and cancer primarily concentrates on renal cancer, esophageal squamous cell carcinoma, breast cancer, among others. Studies reveal that KCNJ15 is significantly downregulated in renal cell carcinoma (RCC), and this reduced expression serves as an independent poor prognostic factor for clear cell RCC (ccRCC). *In vitro* experiments demonstrate that overexpression of KCNJ15 inhibits RCC cell proliferation and induces cell cycle arrest by upregulating the expression of p21 protein. Simultaneously, overexpression of KCNJ15 leads to the downregulation of N-cadherin, vimentin, and MMP-7 protein expression, indicating that KCNJ15 can hinder RCC cell migration and invasion by suppressing the transition of epithelial cells to mesenchymal cells and downregulating MMP-7 (Liu et al., 2019). Nakamura et al. (2020) discovered that the expression level of KCNJ15 in esophageal squamous cell carcinoma (ESCC) cell lines exhibits significant variation, and high expression of KCNJ15 serves as an independent poor prognostic factor for ESCC. The results of PCR array analysis indicated that COL3A1, JAG1, and F11R mRNA exhibit a positive correlation with KCNJ15. Previous studies have shown that COL3A1, Jag1 and F11R are involved in tumor progression through the PI3K/AKT signaling pathway (Zhang M. et al., 2024; Tan et al., 2012; Tian et al., 2015; Nava et al., 2011), which also mediates the epithelial-mesenchymal transition (EMT) process (Xu et al., 2015). Evidently, KCNJ15 may play a role in cancer progression by regulating the cell cycle and inhibiting the EMT process, indicating that KCNJ15 could potentially serve as a target for cancer therapy.

Cell migration is the fundamental process of tumor metastasis. The primary function of the integrin family is to anchor cells to the extracellular matrix (ECM), thus playing a crucial role in cell migration. α9β1 integrin accelerates cell migration by binding its cytoplasmic domain to spermidine/spermine acetyltransferase (SSAT), which enhances α9-mediated migration through the catabolic metabolism of spermidine and/or spermine (deHart et al., 2008; Chen et al., 2004). Given that spermine and spermidine can function as physiological blockers of Kir channels, it is hypothesized that the Kir family may be involved in the process by which α9β1 integrin promotes cell metastasis (Ficker et al., 1994). Veeravalli et al. (2012) discovered that shRNA-mediated double knockdown of MMP-9 and uPAR/cathepsin B leads to the downregulation of both mRNA and protein levels of SSAT. Furthermore, knockdown of SSAT in glioma xenograft cell lines (4,910 and 5,310) significantly decreased their migration ability. Subsequent research revealed that treating 4,910 and 5,310 cells, which overexpressed MMP-9 and uPAR/cathepsin B, with barium or Kir4.2 siRNA significantly inhibited their migration ability. This suggests that knockdown of Kir4.2 can suppress glioma cell migration mediated by MMP-9 and uPAR/cathepsin B. Additionally, in glioma xenograft cell lines (4,910 and 5,310), colocalization of α9 and Kir4.2 was observed. However, double knockdown of MMP-9 and uPAR/cathepsin B significantly reduced the colocalization of α9 and Kir4.2 (Veeravalli et al., 2012). These findings underscore the necessity of Kir4.2 for α9β1 integrin-mediated glioma metastasis, hinting at the potential significance of KCNJ15 in tumor metastasis.

Chemotherapy stands as a crucial method for treating malignant tumors; however, chemoresistance poses a significant challenge in cancer therapy (Delou et al., 2019). Lysosomes, organelles within cells featuring a single-layer membrane sac structure, contain various hydrolytic enzymes such as phosphatase, lipase, protease, nuclease, glycosidase, and sulfatase, capable of nonspecifically degrading intracellular macromolecules (Zhang et al., 2021). Lysosomes play a pivotal role in regulating tumor cell proliferation, invasion, and the tumor microenvironment. Dysfunction in lysosomes can lead to drug redistribution, ultimately resulting in drug resistance and a poor prognosis for tumor patients. Located on the surface of lysosomes, V-ATPase functions as a proton pump, maintaining the stability of lysosomal pH. Studies have revealed that the specific expression of KCNJ15 decreases in triple negative breast cancer, particularly in paclitaxel-resistant cells of this type. Furthermore, patients with low KCNJ15 expression exhibit a shorter Overall Fraction Survivor (OFS) compared to those with high KCNJ15 expression. KCNJ15 can bind to the V-ATPase subunit ATP6V0A1, facilitating the separation of V0 and V1 subunits within V-ATPase. This interaction inhibits the proton pump effect of V-ATPase, resulting in lysosomal dysfunction, which subsequently mediates chemoresistance. Conversely, small molecule drugs (CMA/BAF) can reverse drug resistance by disrupting the binding between KCNJ15 and V-ATPase (Qiao et al., 2023). Evidently, KCNJ15 promotes the development of chemotherapy resistance in breast cancer by influencing lysosomal function. It may serve as a predictor for pre-chemotherapy resistance in breast cancer and emerge as a potential target for treating drug-resistant breast cancer.

### 4.4 Kir4.2 and other diseases

The retinal pigment epithelium (RPE) is a fundamental component of the retina, connecting Bruch’s membrane and the choroid on the lateral side, and the outer segment of photoreceptor cells on the medial side. It plays an indispensable role in maintaining visual function (Strauss, 2005). Consequently, impairments in the structure and function of the RPE will lead to various retinal diseases, including retinitis pigmentosa (RP), age-related macular degeneration (AMD), and Stargardt disease (STGD). K^+^ channels located in the apical membrane of RPE cells mediate the spatial buffering of K^+^ concentration beneath the retina, maintain the resting membrane potential, and support the functions of the Na^+^-K^+^ pump and Na^+^-K^+^-2Cl^-^ pump cotransporters (Hughes and Takahira, 1996). RPE cells express a variety of Kir channel subtypes. Reverse transcription polymerase chain reaction (RT-PCR) analysis reveals that, besides Kir7.1, seven other Kir channel subunits (Kir1.1, Kir2.1, Kir2.2, Kir3.1, Kir3.4, Kir4.2, and Kir6.1) are also expressed in RPE cells (Yang et al., 2008). Studies indicate that both hypoxia and extracellular hypertonia can stimulate the expression and secretion of vascular endothelial growth factor (VEGF). Exogenous VEGF can lead to a reduction in Kir4.2 gene expression in RPE cells, and this effect can be inhibited by the selective blocker of VEGF receptor-2 (SU1498). It is evident that VEGF can decrease the expression of Kir4.2 gene under conditions of hypoxia and extracellular hypertonia, and siRNA-mediated knockdown of Kir4.2 can lead to a reduction in RPE cell viability and proliferation (Beer et al., 2022). Based on the aforementioned studies, the Kir4.2 channel plays a crucial role in maintaining the survival and proliferation abilities of RPE cells, indicating that KCNJ15 holds significant importance in the development of retinal diseases.

Ankylosing spondylitis (AS) and ulcerative colitis (UC) share similarities in terms of incidence and pathogenesis. Comprehensive bioinformatics analysis showed that the KCNJ15 gene is a common diagnostic marker for both AS and UC, with the oxidative phosphorylation pathway being a commonly enriched pathway for both diseases. Both AS and UC are immune-mediated chronic inflammatory diseases. The CIBERSORT results have highlighted a significant correlation between KCNJ15 and immune infiltrating cells, indicating the potential value of KCNJ15 in the diagnosis and treatment of AS and UC (Zhou et al., 2023). Another comprehensive bioinformatics analysis revealed that KCNJ15, along with TSPYL5, PARVG, RTN1, CTSW, HMOX1, DCAF12L1, VNN2, and ANXA1, were identified as potential prognostic predictive genes for endometrial cancer (UCEC) and polycystic ovary syndrome (PCOS). Additionally, KCNJ15, TSPYL5, RTN1, HMOX1, DCAF12L1, VNN2, and ANXA1 were found to be associated with survival time, tumor mutation burden (TMB), and immune infiltration in UCEC (Wu et al., 2023). These findings imply that KCNJ15 plays a role in the development of various human diseases. Further research is warranted to validate its significance, aiming to identify new targets for disease diagnosis and treatment.

## 5 Conclusion

This article primarily introduces the key physiological characteristics of the Kir4.2 channel, the pathophysiological processes associated with it, and its correlation with human diseases. The activation of the Kir4.2 channel relies on $K_{0}^{+}$ . In the kidney, the Kir4.2 channel functions as a potassium sensor, contributing to the maintenance of electrolyte homeostasis and acid-base balance. Additionally, it mediates renal damage caused by low potassium levels and polymyxin-induced nephrotoxicity. The Kir4.2 channel exhibits pHi sensitivity. However, unlike Kir1.1, the pHi sensitivity of the Kir4.2 channel is not coupled with $K_{0}^{+}$ sensitivity. The Kir4.2 protein can interact with intracellular polyamines, enabling it to sense extracellular electric fields and subsequently induce the directional migration of cells. Knockdown of KCNJ15 decreased histamine-stimulated acid secretion in rabbit primary gastric parietal cells; however, the precise mechanism remains to be further investigated. As a susceptibility gene for T2DM, KCNJ15 has yielded contradictory conclusions in existing studies, necessitating further research to ascertain its clinical significance in T2DM. Additionally, KCNJ15 gene mutations are implicated in the progression of neurological diseases, such as Alzheimer’s disease and epilepsy. Cancer research has revealed that KCNJ15, as a differentially expressed gene, correlates with the clinical prognosis of renal cancer, esophageal squamous cell carcinoma, breast cancer, and glioma (Table 1). KCNJ15 may contribute to cancer occurrence, migration, and drug resistance by engaging in the EMT process, regulating the cell cycle, and disrupting lysosomal function. Furthermore, Kir4.2 protein is expressed in RPE cells, and knockdown of Kir4.2 diminishes the viability and proliferative capacity of RPE cells, indicating the potential value of Kir4.2 in retinal diseases.

**TABLE 1:** The function/role of Kir4.2 in human diseases.

Disease	Function/Role
Epilepsy	The epilepsy-related SNP rs2833098 may regulate the expression level of KCNJ15 in human temporal lobe brain tissue and serve as an epilepsy risk biomarker (Wang et al., 2022)
Clear cell renal cell carcinoma (ccRCC)	KCNJ15 inhibits the proliferation of RCC cells by upregulating the expression of P21 protein, induces cell cycle arrest, and may inhibit the migration and invasion of RCC cells by suppressing epithelial-mesenchymal transition (Liu et al., 2019)
Esophageal squamous cell carcinoma (ESCC)	High expression of KCNJ15 is an independent poor prognostic factor for ESCC, potentially contributing to cancer progression by influencing epithelial-mesenchymal transition (Nakamura et al., 2020)
Triple negative breast cancer (TNBC)	KCNJ15 promotes the occurrence of breast cancer chemotherapy resistance by affecting lysosomal function (Qiao et al., 2023)
Glioma	Knockdown of Kir4.2 suppresses MMP-9 and uPAR/cathepsin B-mediated glioma cell migration (Veeravalli et al., 2012)
Type 2 diabetes mellitus	There is a significant correlation between the KCNJ15-related SNP rs3746876-T and type 2 diabetes mellitus (T2DM) (Okamoto et al., 2010; Fukuda et al., 2013; Okamoto et al., 2012)
Alzheimer’s disease	The variation at the SNP rs928771 locus associated with KCNJ15 affects the age of onset of AD, with a small number of allele carriers experiencing earlier onset (Zhou et al., 2018)

The physiological functions and characteristics of the Kir4.2 channel are gradually being uncovered, while its significance in human diseases awaits further confirmation through research. Currently, the absence of specific inhibitors for the Kir4.2 channel restricts our ability to delve deeper into its physiological functions. Consequently, future studies will focus on developing inhibitors for the Kir4.2 channel, enhancing our comprehensive understanding of it. Additionally, research into the mechanism of Kir4.2 in human diseases has identified it as a potential target for targeted therapy.
